# Impact of the lockdown on acute stroke treatments during the first surge of the COVID-19 outbreak in the Netherlands

**DOI:** 10.1186/s12883-021-02539-4

**Published:** 2022-01-11

**Authors:** Faysal Benali, Lotte J. Stolze, Anouk D. Rozeman, Wouter Dinkelaar, Jonathan M. Coutinho, Bart J. Emmer, Rob A. R. Gons, Lonneke F. S. Yo, Julia H. van Tuijl, Issam Boukrab, Dianne H. K. van Dam-Nolen, Ido R. van den Wijngaard, Geert J. Lycklama à Nijeholt, Karlijn F. de Laat, Lukas C. van Dijk, Heleen M. den Hertog, H. Zwenneke Flach, Marieke J. H. Wermer, Marianne A. A. van Walderveen, Paul J. A. M. Brouwers, Tomas Bulut, Sarah E. Vermeer, Marie Louise E. Bernsen, Maarten Uyttenboogaart, Reinoud P. H. Bokkers, Jeroen D. Boogaarts, Frank-Erik de Leeuw, H. Bart van der Worp, Irene C. van der Schaaf, Wouter J. Schonewille, Jan A. Vos, Michel J. M. Remmers, Farshad Imani, Diederik W. J. Dippel, Wim H. van Zwam, Paul J. Nederkoorn, Robert J. van Oostenbrugge

**Affiliations:** 1 Department of Neurology and Radiology & Nuclear Medicine, Maastricht University Medical Center, Maastricht, the Netherlands; 2 Department of Neurology and Radiology, Amsterdam UMC, Amsterdam, the Netherlands; 3 Department of Neurology and Radiology, Albert Schweitzer Hospital, Dordrecht, the Netherlands; 4 Department of Neurology and Radiology, Catharina Hospital, Eindhoven, the Netherlands; 5 Department of Neurology and Radiology, Elisabeth TweeSteden Hospital, Tilburg, the Netherlands; 6 Department of Neurology and Radiology, Erasmus University Medical Center, Rotterdam, the Netherlands; 7 Department of Neurology and Radiology, Haaglanden Medical Center, The Hague, the Netherlands; 8 Department of Neurology and Radiology, Haga Hospital, The Hague, the Netherlands; 9 Department of Neurology and Radiology, Isala Hospital, Zwolle, the Netherlands; 10 Department of Neurology and Radiology, Leiden University Medical Center, Leiden, the Netherlands; 11 Department of Neurology and Radiology, Medisch Spectrum Twente, Enschede, the Netherlands; 12 Department of Neurology and Radiology, Rijnstate Hospital, Arnhem, the Netherlands; 13 Department of Neurology and Radiology, University Medical Center Groningen, Groningen, the Netherlands; 14 Department of Neurosurgery and Neurology, Radboud University Nijmegen Medical Center, Nijmegen, the Netherlands; 15 Department of Neurology and Neurosurgery and Radiology, University Medical Center Utrecht, Utrecht, the Netherlands; 16 Department of Neurology and Radiology, Sint Antonius Hospital, Nieuwegein, the Netherlands; 17 Department of Neurology and Radiology, Amphia Hospital, Breda, the Netherlands

**Keywords:** COVID-19, Lockdown, Acute stroke care, Intravenous thrombolytics, Endovascular thrombectomy, NIHSS

## Abstract

**Introduction:**

We investigated the impact of the Corona Virus Disease 2019 (COVID-19) pandemic and the resulting lockdown on reperfusion treatments and door-to-treatment times during the first surge in Dutch comprehensive stroke centers. Furthermore, we studied the association between COVID-19-status and treatment times.

**Methods:**

We included all patients receiving reperfusion treatment in 17 Dutch stroke centers from May 11th, 2017, until May 11th, 2020. We collected baseline characteristics, National Institutes of Health Stroke Scale (NIHSS) at admission, onset-to-door time (ODT), door-to-needle time (DNT), door-to-groin time (DGT) and COVID-19-status at admission. Parameters during the lockdown (March 15th, 2020 until May 11th, 2020) were compared with those in the same period in 2019, and between groups stratified by COVID-19-status. We used nationwide data and extrapolated our findings to the increasing trend of EVT numbers since May 2017.

**Results:**

A decline of 14% was seen in reperfusion treatments during lockdown, with a decline in both IVT and EVT delivery. DGT increased by 12 min (50 to 62 min, *p*-value of < 0.001). Furthermore, median NIHSS-scores were higher in COVID-19 - suspected or positive patients (7 to 11, p-value of 0.004), door-to-treatment times did not differ significantly when stratified for COVID-19-status.

**Conclusions:**

During the first surge of the COVID-19 pandemic, a decline in acute reperfusion treatments and a delay in DGT was seen, which indicates a target for attention. It also appeared that COVID-19-positive or -suspected patients had more severe neurologic symptoms, whereas their EVT-workflow was not affected.

**Supplementary Information:**

The online version contains supplementary material available at 10.1186/s12883-021-02539-4.

## Introduction

On March 11th, 2020, the World Health Organization (WHO) characterized the outbreak of the Corona Virus Disease 2019 (COVID-19) as a pandemic [1]. On March 15th, 2020, the Dutch government implemented a nationwide lockdown in which people were advised to stay home and keep social distance [2]. These rules were maintained for nearly two months until May 11th, when several restrictions were abated. At the peak of the first COVID-19 wave there were 65 daily new confirmed COVID-19 cases per day per million inhabitants and 189 weekly COVID-19 hospital admissions per million inhabitants; compared to 71 daily cases and 317 hospital admissions per million in the United Kingdom, and 96 daily cases per million in the United States [3].

During the pandemic, impact on stroke services and a reduction in the number of acute stroke admissions were reported in several countries [4]. Health care workers had to follow strict protection measures, which may have affected acute stroke workflows in hospitals [5, 6], especially in COVID-19-positive (or suspected) acute ischemic stroke (AIS)-patients. It remains unclear whether the reported delays and decline in stroke admissions have also led to nationwide declines in the number of AIS-patients treated with reperfusion therapy, either intravenous thrombolytics (IVT) and/or endovascular treatment (EVT), in the Netherlands, and if previous door-to-treatment times could be maintained for COVID-19-positive (or suspected) patients.

Acute care for AIS-patients has progressed considerably in the past decade. In 2015 the MR CLEAN trial demonstrated a beneficial effect of EVT for patients with an arterial occlusion in the anterior circulation within 6 h after onset of symptoms [7]. These findings were replicated in 4 subsequent RCTs [8] and lead to the approval of endovascular therapy as standard treatment by the Dutch government in 2017 [9]. In recent years, several studies have demonstrated a possibility to treat patients beyond the conventional 4.5 h after stroke onset for IVT [10, 11], and 6 h for EVT [12, 13] using additional (perfusion) imaging. All these advances have led to an increased number of patients considered eligible for acute reperfusion therapies.

Our aim was to assess in Dutch comprehensive stroke centers whether the number of AIS-patients treated with reperfusion therapy changed during the lockdown period of the first COVID-19 surge, and whether the door-to-treatment times were altered. We did so by using nationwide data and taking trends of EVT numbers since 2017 into consideration. Lastly, we also examined the differences in door-to-treatment times when stratifying the studied population into COVID-19-suspected and –positive, and unsuspected patients.

## Materials and methods

### Study design and data collection

We included all consecutive AIS-patients, aged 18 years or older, who received IVT, EVT or both in one of the 17 comprehensive stroke centers in the Netherlands, from May 11th, 2017 until May 11th, 2020. The participating centers, outside which EVT is not performed, collaborate in the MRCLEAN registry [14]. Patient data from 2017 to 2019 were extracted from the Dutch Acute Stroke Audit (DASA) register [15], a nationwide prospective quality audit, including acute stroke patients in the Netherlands. The data of 2020 were directly collected from the participating centers and were registered in a fully protected university-based online platform (SharePoint, Office 365). The collected data entails exclusively information about treatments given within the participating thrombectomy capable hospitals; no data were collected on IVT given in primary (non-) stroke centers. No ethical approval or patient informed consent was required for this study.

We collected the following patient characteristics: age, sex, National Institutes of Health Stroke Scale (NIHSS) at presentation, and onset-to-door time (ODT). Regarding treatment information, the type of reperfusion treatment (IVT and/or EVT), door-to-needle time (DNT), and the door-to-groin time (DGT) were collected. Additional information on (possible) COVID-19 infection at presentation of the AIS-patients presenting during the lockdown period (March 15th, 2020 until May 11th, 2020) was gathered.

First, the number of IVT and EVT treatments, patient characteristics (age, sex, NIHSS at presentation, ODT), and door-to-treatment times (DNT, DGT) during the lockdown period (March 15th, 2020 until May 11th, 2020) were compared to the same period in 2019, on a national and regional level. We subdivided the included hospitals into four regions (see supplemental Table 1 and supplemental Fig. 1). This chosen subdivision aimed to reflect the geographical location, the region’s population density, size of the included population in the included hospitals, and the severity by which the region was affected by the COVID-19 epidemic. As a proxy to illustrate the severity of the hospitals’ additional workload per region, the total number of COVID-19 admissions [16] per 10,000 inhabitants [17] was calculated. The number of COVID-19 admissions was chosen instead of the number of positive testing since testing was minimal during the first surge in the Netherlands. To take the increasing number of EVTs over the recent years into consideration, the expected number of EVTs during two lockdown months (March 12th until April 11th and April 12th until May 11th, 2020) was compared with the monthly observed numbers during that period. The expected number was based on a regression analysis of the monthly numbers of EVTs from May 12th, 2017 until March 11th, 2020.

Secondly, patients presented during the defined lockdown period were stratified by COVID-status. COVID-19-positive and COVID-19-suspected patients were pooled together (since both were expected to have a similar effect on door-to-treatment times) and compared to patients who were not suspected of having COVID-19 or who already had a negative polymerase chain reaction (PCR) test after nasopharyngeal swab at presentation. Suspicion of having COVID-19 was based on clinical (cough, fever, malaise, muscle pain) and/or radiological features (detected on chest CT-scan). COVID-19-positive patients had a positive PCR-test after nasopharyngeal swab at presentation. Possible differences between the groups were analyzed for patient characteristics and door-to-treatment times.

### Statistical analysis

All parameters were checked on skewness - kurtosis and plotted in histograms. When normally distributed, a chi-squared test was used for binary variables and t-test for continuous variables. For non-normally distributed parameters, the Kruskal-Wallis test was used for categorical variables and a Mann-Whitney-U test for continuous variables. The absolute numbers of IVTs and EVTs were compared using an exact test for Poisson rates. The expected number of EVTs in the lockdown months was based on linear regression analyses of previous months (up to May 12th, 2017). Statistical analysis was performed in RStudio Version 1.3.959 [18].

## Results

Five hundred twenty-six patients received acute reperfusion therapy during the lockdown period; 293 patients were treated with EVT and 317 with IVT. Six hundred fifteen patients were treated in the reference period in 2019; 305 patients were treated with EVT (*p*-value of 0.653) and 355 with IVT (p-value of 0.153). Baseline characteristics between the lockdown period and the same period in 2019 do not appear to be changed. Median NIHSS was slightly higher compared to the reference period in 2019 (8 vs. 7, p-value of 0.014). The DGT of 62 min in the lockdown period was significantly longer than the DGT of 50 min in the reference period (p-value of < 0.001) (see Table 1). The observed increased DGT was observed in all regions (see Supplemental Tables 1 and 2). It should, however, be noted that during the lockdown period more data were missing compared to the reference period.

**Table 1:** All treated AIS-patients from March 15th until May 11th in 2020 (lockdown) and 2019 (reference)

	2019 (***n*** = 615)		2020 (***n*** = 526)		***P***-value
**Number of IVT (N)**	355		317		0.153
**Number of EVT (N)**	305		293		0.653
		missing		missing	
**Age, mean (SD)**	72 (13)	4.7%	71 (13)	4.2%	0.079
**Female sex (%)**	47.7%	5.5%	42.8%	4.0%	0.119
**NIHSS, median (IQR)**	7 (3–14)	17.9%	8 (4–16)	10.5%	**0.014**
**ODT, median (IQR)**^a^	96 (52–151)	18.4%	94 (53–157)	32.3%	0.913
**DNT, median (IQR)**^a^	27 (20–40)	2.0%	30 (20–42)	18.3%	0.052
**DGT, median (IQR)**^a^	50 (27–73)	10.2%	62 (40–86)	20.5%	**< 0.001**

^a^All times are displayed in minutes

**Fig. 1:**
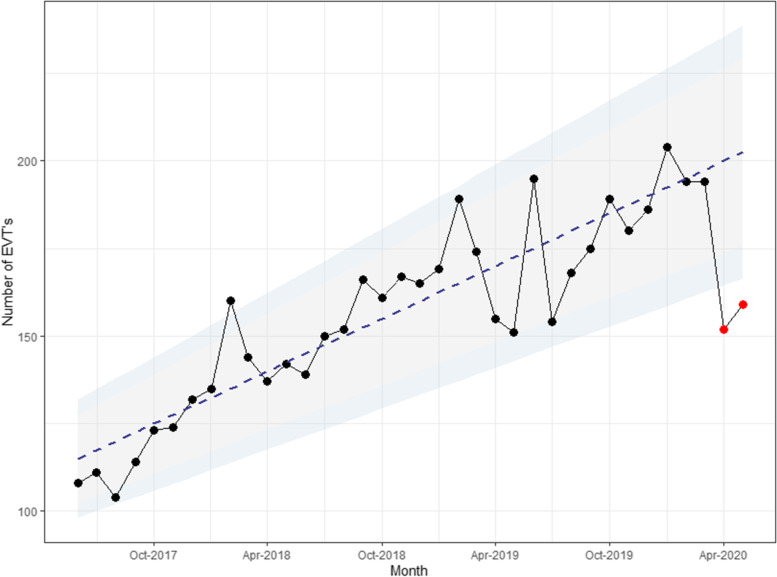
Number of EVTs per month of all Dutch comprehensive stroke centers from May 12th, 2017 until May 11th, 2020. The red dots represent the lockdown months. The grey and blue area represent the 95 and 99% confidence interval of the regression line (based on the non-COVID months) respectively. The monthly numbers are based on the period ranging from the 12th of the previous month to the 11th of the mentioned month; i.e. October 2018 includes data from September 12th, 2018 until October 11th, 2018

In the Netherlands, the monthly EVT numbers showed an increase in observed cases since 2017, with a rather profound decline during the period of April to May 2020 (152 and 159 cases were observed compared to the expected numbers of 200 and 202, respectively). These numbers even declined below 95% (April 173–226 cases; May 176–229 cases) and 99% confidence intervals (April 164–236 cases; May 166–239 cases; see Fig. 1).

A total of 494 patients were included in the COVID-19-sub analysis, of whom 427 patients were not suspected or were already tested negative, while 67 patients (14%) were suspected of having COVID-19 infection or were already tested positive at presentation. One hospital (*n* = 32) did not provide information on COVID19-suspicion or PCR test results and was excluded from this analysis. Median NIHSS score at presentation for COVID-19-positive/−suspected patients was significantly higher than for the COVID-19-negative/unsuspected group (7 versus 11, *p*-value of 0.004). Door-to-treatment times (DNT, DGT) were similar between the two groups (Table 2).

**Table 2:** Treated AIS-patients from March 15th until May 11th, 2020 (lockdown) stratified by COVID-19-status at presentation

	COVID-19-negative or not-suspected at presentation (***n*** = 427)		COVID-19-positive or suspected at presentation (***n*** = 67)		***P***-value
		missing		missing	
**Age, mean (SD)**	71 (13)	0.2%	73 (13)	0%	0.188
**Female sex (%)**	42.6%	0.0%	41.8%	0%	1.000
**NIHSS, median (IQR)**	7 (3–15)	7.3%	11 (7–19)	3.0%	**0.004**
**ODT, median (IQR)**^a^	95 (52–154)	30.9%	81 (56–177)	25.4%	0.774
**DNT, median (IQR)**^a^	30 (20–42)	12.1%	30 (20–38)	13.5%	0.632
**DGT, median (IQR)**^a^	62 (39–83)	22.4%	59 (44–81)	15.6%	0.775

^a^All times are displayed in minutes

## Discussion

The lockdown period during the first surge of the COVID-19 epidemic in the Netherlands resulted in a decline of 14% in patients with AIS treated with reperfusion treatments in comprehensive stroke centers. As for the IVT treatments, a decline of 11% was observed (p-value of 0.153), while EVT treatments declined by 4% (p-value of 0.653). The discrepancy between the observed IVT and EVT decline, could hypothetically be explained by the fact that patients with minor stroke symptoms were less likely to present at a hospital (within treatment-windows) while patients with more severe stroke symptoms presented to the hospital within the treatment window, yet in slightly lower numbers compared to 2019. Our results also show an absolute decrease in monthly EVT cases, when taking the increasing trend of EVT numbers since 2017 into consideration. We did not include a trend analysis for IVT numbers since these numbers have been stable over the last years (according to the data in the DASA).

The COVID-19 epidemic could hypothetically have overlapped with a period of deceleration in the growth in EVT numbers. However, the fact that the studied COVID-19 months have deviated from the analyzed trend outside the 99%-confidence interval makes it unlikely that such deceleration explains the total decline (see Fig. 1). This is similar to other studies [ 19–22]. Most earlier studies on EVT numbers during the first COVID-19 wave were, however, unlike ours, not nationwide and did not include trends going back to 2017 [19, 23–25].

Onset-to-door times for patients receiving reperfusion therapy did not change significantly during the lockdown period. However, we have no data on patients who arrived outside the recommended time window for reperfusion therapy since they are not included in this study (because they did not receive reperfusion therapy). Furthermore, while the median door-to-needle time was slightly increased during the lockdown period compared to the 2019-reference period (30 and 27 min respectively, *p*-value of 0.052), the door-to-groin time showed a significant increase of 12 min (50 to 62 min, p-value of < 0.001). It could be argued that this prolongation is due to an increased proportion of patients receiving IVT subsequently followed by EVT (8% in 2019 vs. 16% in 2020). However, lengthened DGT was also observed in the subgroup of patients having received only EVT (46 to 58 min, p-value of 0.001) and the subgroup of patients who received both treatments (66 to 70 min, p-value of 0.651). The differences in the prolongation of the DGT between the two subgroups (only EVT and both treatments) could possibly be explained by a shift in the proportion of patients who presented primarily to an EVT-center (mothership), or more extensive (COVID-19) work-up before an EVT-procedure that could take place during IVT in the subgroup receiving both treatments. Nonetheless, these results show us that the workflows surrounding IVT appeared to be unaffected, while workflows of EVTs were prolonged during the lockdown period. Evaluating in more detail these prolonged EVT-workflows seems thus of great importance in maintaining optimal acute ischemic stroke care.

The observed prolonged DGT during the lockdown period is in line with a French study [26]. However, our results are contradictory to some findings in a study from Barcelona [20], which showed stability of door-to-treatment times. Our study has nationwide coverage and shows lengthening of DGT in all regions, independently of how severely a region was affected by the COVID-19 epidemic (see Supplemental Tables 1 and 2).

Lastly, when patients were suspected of having COVID-19 or when patients had a positive PCR test, the door-to-treatment times (DNT, DGT) were not significantly altered. On the other hand, COVID-19-positive or suspected COVID-19 patients with AIS presented with more severe symptoms according to the median NIHSS (7 compared to 11, *p*-value of 0.004).

Maintaining the same door-to-treatment times seems remarkable considering possible additional protection measures that had to be taken. In Dutch emergency departments, not all community patients were treated as COVID-19-suspected. Protocols differed for COVID-19 suspected or -positive patients compared to patients without a COVID-19 suspicion or patients already having a negative PCR -test. Higher NIHSS in COVID-19-positive patients matches earlier findings [27] and might be explained by mechanisms of the virus causing large vessel occlusion [28]. However, since only information was gathered about the COVID-19-status of patients at admission, and suspected patients (55 patients) were pooled together with positive tested cases (12 patients), no conclusions on causative relationship between COVID-19-status, appearance of large vessel occlusion and higher NIHSS rates, can be drawn.

One of this study’s important strengths is the large nationwide dataset covering IVT numbers from 2019 and 2020 and EVT numbers from the past 3 years in Dutch stroke centers. This gave us the ability to, in addition to absolute comparisons, measure the impact of the EVT numbers during the lockdown period on a trend line ranging from 2017 to 2020. However, the downside of such a large nationwide data registry is the inherent fact of missing data (compared to for example clinical trials). During the observed period in 2020 more data were missing compared to the same period in 2019. This difference may be explained by the different way the data was collected or by less registration of the variables due to the extra strain on health care departments. In addition, this study only includes patients who received reperfusion therapy. Therefore, the number of patients with a delayed presentation, and thus missing treatment, could not be determined. Also, information on the number of treatment-eligible patients who did not receive treatment and who received IVT in primary non-EVT centers were not available. This restricted us in presenting valid results on regional differences. Despite these limitations, we believe that the trends and suggestions derived from this study still provide valid conclusions and starting points for further research, and tools for maintaining quality care during periods of crisis.

## Conclusions

Reperfusion treatments during the Dutch lockdown in the first wave of the COVID-19 pandemic declined by 14%; IVT cases declined by 11% and EVT cases declined by 4%. When the trend of increasing numbers of EVTs since May 2017 was taken into account, the number of observed cases was significantly lower than expected. These findings should encourage healthcare providers and administration to actively work on maintaining optimal accessibility of acute stroke care and motivate patients and relatives to seek medical attention when stroke symptoms occur. Stroke awareness campaigns are, however, absolutely needed to avoid that patients with mild symptoms refrain from seeking medical care. Thereby, our study encourages to further evaluate EVT-workflows in order to maintain optimal acute stroke care during a healthcare crisis. On the other hand, since COVID-19-positive or suspected AIS-patients showed more severe neurologic symptoms without prolonged door-to-treatment times, we show that it is possible to maintain good workflows even if protective measures have to be taken.

## Supplementary Information

**Additional file 1: Supplemental table 1**. Subdivision in regions. *Total number of new COVID-19 hospital admissions in all hospital in a region from March 15th, 2020 until May 11th, 2020, based on data from the Dutch public health service (GGD)16 and Statistics Netherlands (CBS)17. This illustrates the severity of crowding due to COVID-19 in a region. **Supplemental figure 1.** Map of the different regions with corresponding EVT-centers. *Total number of new COVID-19 hospital admissions in all hospital in a region from March 15^th^, 2020 until May 11^th^, 2020, based on data from the Dutch public health service (GGD) [16] and Statistics Netherlands (CBS) [17]. This illustrates the severity of crowding due to COVID-19 in a region. **Supplemental table 2.** All treated AIS-patients from March 15^th^ until May 11^th^, 2020 (lockdown) and 2019 (reference), subdivided in regions. * All times are displayed in minutes.

## Data Availability

The datasets used and/or analyzed during the current study are available from the corresponding author on reasonable request.

## Abbreviations

WHO: World Health Organization
COVID-19: Corona Virus Disease 19
AIS: Acute Ischemic Stroke
EVT: Endovascular Thrombectomy
IVT: Intravenous Thrombolysis
DASA: Dutch Acute Stroke Audit
NIHSS: National Institutes of Health Stroke Scale
ODT: Onset to Door Time
DNT: Door to Needle Time
DGT: Door to Groin Time
PCR: Polymerase Chain Reaction
