# Supplementary material for: Impact of the lockdown on acute stroke treatments during the first surge of the COVID-19 outbreak in the Netherlands
Source: BMC Neurol. 2022 Jan 11;22:22. doi: 10.1186/s12883-021-02539-4 (PMC8749107; doi:10.1186/s12883-021-02539-4)
Supplement: Supplementary file 1 — Additional file 1: Supplemental table 1. Subdivision in regions. *Total number of new COVID-19 hospital admissions in all hospital in a region from March 15th, 2020 until May 11th, 2020, based on data from the Dutch public health service (GGD)16 and Statistics Netherlands (CBS)17. This illustrates the severity of crowding due to COVID-19 in a region. Supplemental figure 1. Map of the different regions with corresponding EVT-centers. *Total number of new COVID-19 hospital admissions in all hospital in a region from March 15th, 2020 until May 11th, 2020, based on data from the Dutch public health service (GGD) [16] and Statistics Netherlands (CBS) [17]. This illustrates the severity of crowding due to COVID-19 in a region. Supplemental table 2. All treated AIS-patients from March 15th until May 11th, 2020 (lockdown) and 2019 (reference), subdivided in regions. * All times are displayed in minutes. [file 12883_2021_2539_MOESM1_ESM.docx]

**Additional files.**

| **Region** | **Hospitals** | **Total number of COVID-19 admissions per 10,000 inhabitants*** |
| --- | --- | --- |
| **Center/East** | University Medical Center Utrecht, Sint Antonius Hospital, Radboud University Medical Center, Rijnstate Hospital | 6.2 |
| **North** | Isala Hospital, University Medical Center Groningen, Medisch Spectrum Twente | 3.7 |
| **South** | Amphia Hospital, Catharina Hospital, Elisabeth TweeSteden Hospital, Maastricht University Medical Center | 11.1 |
| **West** | Leiden University Medical Center, Haaglanden Medical Center, Haga Hospital, Albert Schweitzer Hospital, Amsterdam UMC, Erasmus University Medical Center | 5.3 |

**Supplemental table 1.** Subdivision in regions. *Total number of new COVID-19 hospital admissions in all hospital in a region from March 15^th^, 2020 until May 11^th^, 2020, based on data from the Dutch public health service (GGD)^16^ and Statistics Netherlands (CBS)^17^. This illustrates the severity of crowding due to COVID-19 in a region.


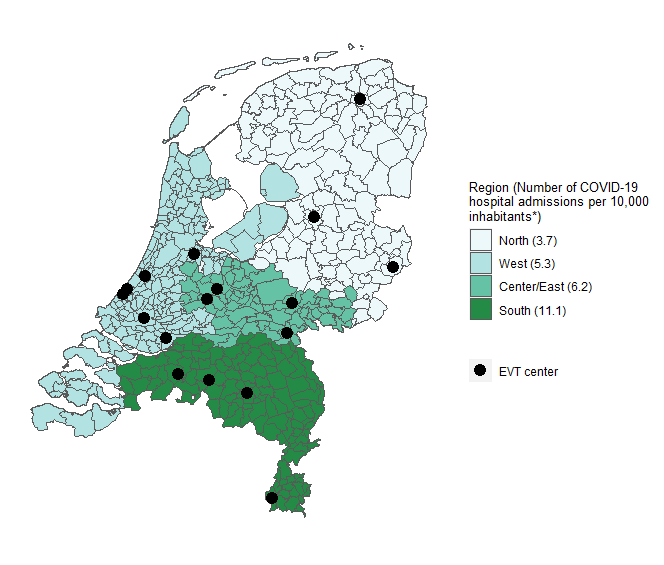


**Supplemental figure 1.** Map of the different regions with corresponding EVT-centers. *Total number of new COVID-19 hospital admissions in all hospital in a region from March 15^th^, 2020 until May 11^th^, 2020, based on data from the Dutch public health service (GGD)^16^ and Statistics Netherlands (CBS)^17^. This illustrates the severity of crowding due to COVID-19 in a region.

|  | **region** | **2019**  **(n=615)** | | **2020**  **(n=562)** | | **P-value** |
| --- | --- | --- | --- | --- | --- | --- |
| **Number of IVTs** | **Center/East** | 71 | | 65 | | 0.668 |
|  | **North** | 61 | | 55 | | 0.643 |
|  | **South** | 70 | | 86 | | 0.230 |
|  | **West** | 153 | | 111 | | 0.011 |
|  |  |  |  |  |  |  |
| **Number of EVTs** | **Center/East** | 84 | | 61 | | 0.067 |
|  | **North** | 46 | | 54 | | 0.484 |
|  | **South** | 73 | | 73 | | 1.000 |
|  | **West** | 102 | | 105 | | 0.889 |
|  |  |  |  |  |  |  |
|  |  |  | missing |  | missing |  |
| **Age, mean (SD)** | **Center/East** | 73 ( 12 ) | 19.6% | 70 ( 14 ) | 0.0% | 0.120 |
|  | **North** | 74 ( 12 ) | 0.0% | 72 ( 12 ) | 1.1% | 0.231 |
|  | **South** | 72 ( 13 ) | 0.0% | 70 ( 14 ) | 16.3% | 0.229 |
|  | **West** | 71 ( 14 ) | 0.0% | 71 ( 12 ) | 0.0% | 0.980 |
|  |  |  |  |  |  |  |
| **Female sex (%)** | **South** | 50.0% | 0.7% | 36.4% | 16.3% | 0.350 |
|  | **West** | 40.3% | 0.4% | 41.1% | 0.0% | 0.991 |
|  | **Center/East** | 41.2% | 20.9% | 42.1% | 0.0% | 0.163 |
|  | **North** | 55.1% | 1.0% | 46.2% | 0.0% | 0.248 |
|  |  |  |  |  |  |  |
| **NIHSS, median (IQR)** | **Center/East** | 8 ( 4 - 15 ) | 20.9% | 8 ( 4 - 16 ) | 1.8% | 0.639 |
|  | **North** | 6 ( 3 - 14 ) | 25.5% | 8 ( 4 - 14 ) | 18.3% | 0.132 |
|  | **South** | 8 ( 4 - 17 ) | 25.7% | 9 ( 4 - 16 ) | 21.7% | 0.669 |
|  | **West** | 6 ( 2 - 13 ) | 8.2% | 8 ( 3 - 15 ) | 4.2% | 0.036 |
|  |  |  |  |  |  |  |
| **ODT, median (IQR)*** | **Center/East** | 110 ( 58 - 157 ) | 24.3% | 109 ( 58 - 158 ) | 10.5% | 0.821 |
|  | **North** | 88 ( 52 - 120 ) | 24.5% | 105 ( 62 - 190 ) | 15.1% | 0.020 |
|  | **South** | 120 ( 58 - 181 ) | 5.9% | 96 ( 47 - 162 ) | 38.0% | 0.103 |
|  | **West** | 82 ( 50 - 135 ) | 19.3% | 70 ( 46 - 124 ) | 50.0% | 0.264 |
|  |  |  |  |  |  |  |
| **DNT, median (IQR)*** | **Center/East** | 35 ( 27 - 48 ) | 8.5% | 38 ( 29 - 50 ) | 0.0% | 0.416 |
|  | **North** | 22 ( 17 - 31 ) | 0.0% | 26 ( 18 - 52 ) | 16.4% | 0.052 |
|  | **South** | 24 ( 20 - 36 ) | 1.4% | 27 ( 20 - 36 ) | 38.4% | 0.498 |
|  | **West** | 26 ( 18 - 41 ) | 0.0% | 26 ( 20 - 36 ) | 14.4% | 0.826 |
|  |  |  |  |  |  |  |
| **DGT, median (IQR)*** | **Center/East** | 45 ( 26 - 67 ) | 27.4% | 59 ( 35 - 83 ) | 6.6% | 0.019 |
|  | **North** | 67 ( 36 - 93 ) | 4.3% | 78 ( 36 - 102 ) | 13.0% | 0.665 |
|  | **South** | 50 ( 23 - 72 ) | 1.4% | 62 ( 50 - 82 ) | 9.6% | 0.001 |
|  | **West** | 50 ( 27 - 68 ) | 4.9% | 58 ( 46 - 75 ) | 40.0% | 0.055 |

**Supplemental table 2.** All treated AIS-patients from March 15^th^ until May 11^th^, 2020 (lockdown) and 2019 (reference), subdivided in regions. * All times are displayed in minutes
